# The Predictive Nature of Individual Differences in Early Associative Learning and Emerging Social Behavior

**DOI:** 10.1371/journal.pone.0030511

**Published:** 2012-01-23

**Authors:** Bethany C. Reeb-Sutherland, Pat Levitt, Nathan A. Fox

**Affiliations:** 1 Department of Human Development, University of Maryland, College Park, Maryland, United States of America; 2 Zilkha Neurogenetic Institute & Department of Cell and Neurobiology, Keck School of Medicine, University of Southern California, Los Angeles, California, United States of America; Institution of Automation, CAS, China

## Abstract

Across the first year of life, infants achieve remarkable success in their ability to interact in the social world. The hierarchical nature of circuit and skill development predicts that the emergence of social behaviors may depend upon an infant's early abilities to detect contingencies, particularly socially-relevant associations. Here, we examined whether individual differences in the rate of associative learning at one month of age is an enduring predictor of social, imitative, and discriminative behaviors measured across the human infant's first year. One-month learning rate was predictive of social behaviors at 5, 9, and 12 months of age as well as face-evoked discriminative neural activity at 9 months of age. Learning was not related to general cognitive abilities. These results underscore the importance of early contingency learning and suggest the presence of a basic mechanism underlying the ontogeny of social behaviors.

## Introduction

During the first year of life, human infants develop remarkable social skills such as an emerging understanding of others' thoughts and intentions [1]. Although the development of infant social behavior has been well described [2], little is known about the mechanisms of learning that underlie its emergence. One possibility is that the infant's abilities to detect and respond to contingencies in the surrounding environment influences the development of social behavior [3], [4]. It is well established that infants can readily learn and detect social [5], [6] and non-social [7], [8] contingencies within the first months of life and it has been suggested that these basic associative learning mechanisms are involved in the ontogeny of social behavior [9], [10], [11]. This has important implications for the observed heterogeneity found in both typical and atypical social development (e.g., autism spectrum disorder) [12]. For example, perturbations in the mechanisms of associative learning early in life may alter the development and maturation of higher-order social cognition that emerges later. Moreover, early associative learning may serve as a major building block for later development of appropriate social behaviors [4], [13].

Associative learning via classical eyeblink conditioning is an ideal strategy to examine the relations between contingent learning and later social behavior development in human infants. It has been extensively used to examine learning in early infancy [14], [15], [16], [17], [18] and the underlying neural circuitry that supports such early learning is well characterized [19], [20]. For the current study, we hypothesized that individual differences in the rapidity of associative learning via delay eyeblink conditioning would relate to individual differences in social behaviors during the first year of life. We predicted that more rapid associative learning at one month of age would be associated with the extent of expressed social behaviors over the first year of life.

## Results

**Figure 1:**
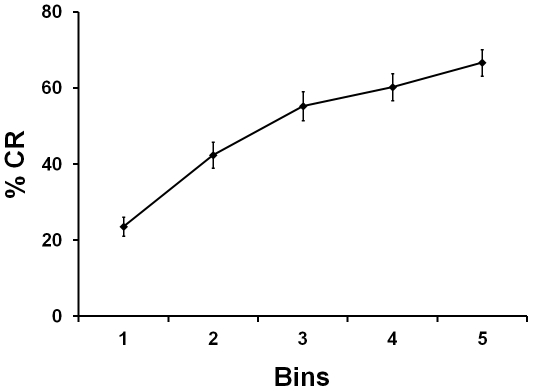
Learning curve for one-month-old infants. Infants learned to associate presentations of a tone with presentations of an airpuff. Error bars represent Mean ± SEM.

At one month of age, infant associative learning was measured using a delay eyeblink conditioning paradigm in which infants were presented with several pairings of a tone followed by a puff of air presented to the eye [17], [18]. Blinks that occurred during presentations of the tone by itself were used to assess infant learning across the experiment. Overall, infants displayed significant learning over the course of conditioning (*F*_4,276_ = 24.75, *P*<.001, Figure 1), thus replicating previous findings examining eyeblink conditioning in young infants [14], [15], [16], [17], [18]. To examine heterogeneity in associative learning, the slope of the learning curve was determined for each infant. Learning slope varied across infants, with some individuals displaying rapid learning and others displaying little to no learning (slope range: −8.3–26.7). This Learning Slope measure was used in subsequent analyses as a predictor to examine the relation between early associative learning and individual differences in tasks specifically designed to examine social and imitative behaviors collected at 5, 9, and 12 months of age and neural activity associated with face discrimination collected at 9 months of age (Table 1).

**Table 1:** Tasks and measures collected at different ages during infancy.

*Age*	*Task*	*Dependent Measure*
*1 month*	Delay Eyeblink Conditioning	Associative Learning
*5 month*	Puppet GamePeek-a-Boo Game	Social ResponsivitySocial Responsivity
*9 month*	Still-Face ParadigmModified Peek-a-Boo GameMother-Stranger ERP TaskImitation Task	Social Contingency Detection Social Contingency Detection Social Discrimination Imitation
*12 month*	Early Social Communication Scale Mullen Early Scales of Learning	Joint Attention General Cognition

**Figure 2:**
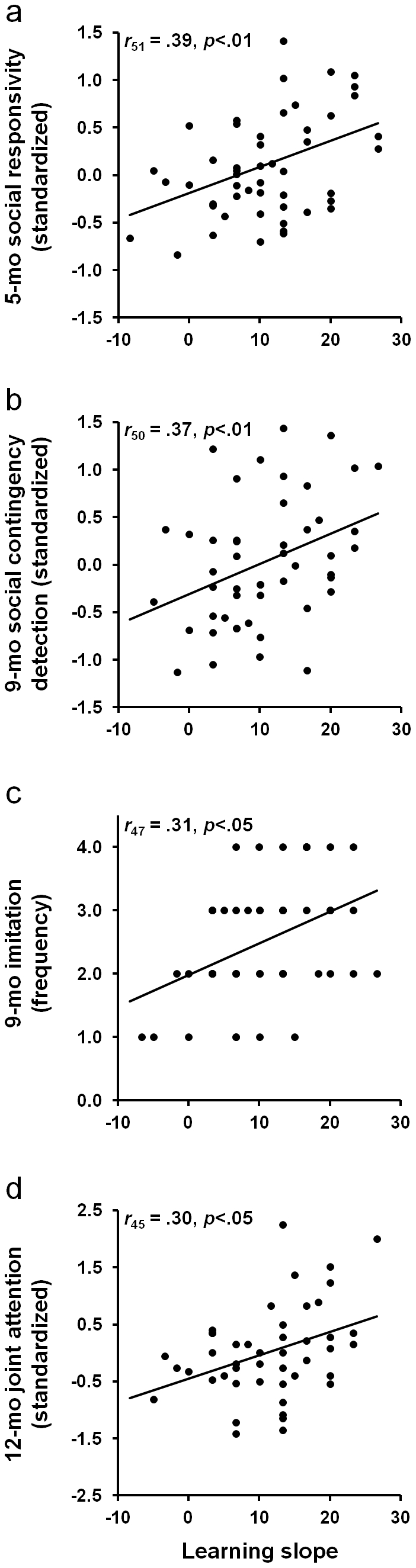
Predictive relation between early learning and social behavior during the first year of life. Individual differences in associative learning measured at 1 month of age were correlated significantly with measures (a) 5-month Social Responsivity, (b) 9-month Social Contingency Detection, (c) 9-month Imitation, and (d) 12-month Joint Attention.

At five months of age, the puppets and peek-a-boo tasks were administered [21]. These tasks are specifically designed to elicit positive contingent responsivity during social interaction. A single Social Responsivity score was computed as the average display of social positivity and social referencing to the mother across the tasks. A remarkable significant correlation between 1-month Learning Slope and 5-month Social Responsivity was found (*r*_51_ = .39, *P*<.01, Figure 2a). This correlation remained even after controlling for maternal report of temperamental positivity (partial *r*_45_ = .36, *P*<.05). Infants who learned more rapidly at one month of age displayed heightened contingent social positivity at 5 months of age.

At 9 months of age, the still-face and modified peek-a-boo tasks were used to examine social contingency detection. Detection of social contingencies in these tasks was measured by examining observed changes in infant behavior following violations of social expectation. A single Social Contingency Detection score was computed, averaged across both tasks. Remarkably, longitudinal stability of the predicted relations between 1-month early associative learning and 9-month social contingency detection was found, with one-month Learning Slope significantly positively correlated with Social Contingency Detection (*r*_50_ = .37, *P*<.01, Figure 2b).

Motor imitation [22] was also assessed at 9 months of age. Infants were presented with four novel age-appropriate tasks which they were allowed to reproduce after a 10-min delay [23], [24]. The frequency of tasks imitated was computed and a significant positive correlation between one-month Learning Slope and Imitation was found (*r*_47_ = .31, *P*<.05, Figure 2c) consistent with infants who learn more rapidly at one month of age imitating more tasks at 9 months of age.

At 12 months of age, infant joint attention, a behavior thought to reflect social understanding of others' minds and behaviors [25], was assessed with the Early Social-Communication Scale (ESCS) [26], a standardized task commonly used to elicit joint attention in infants. A Joint Attention Score was computed and defined as the average amount of initiating joint attention and responding to joint attention exhibited during the ESCS. One-month Learning Slope was found to be significantly correlated with 12-month Joint Attention (*r*_45_ = .30, *P*<.05, Figure 2d) suggesting that rapid associative learning at one month of age was associated with greater amounts of joint attention at 12 months of age.

**Figure 3:**
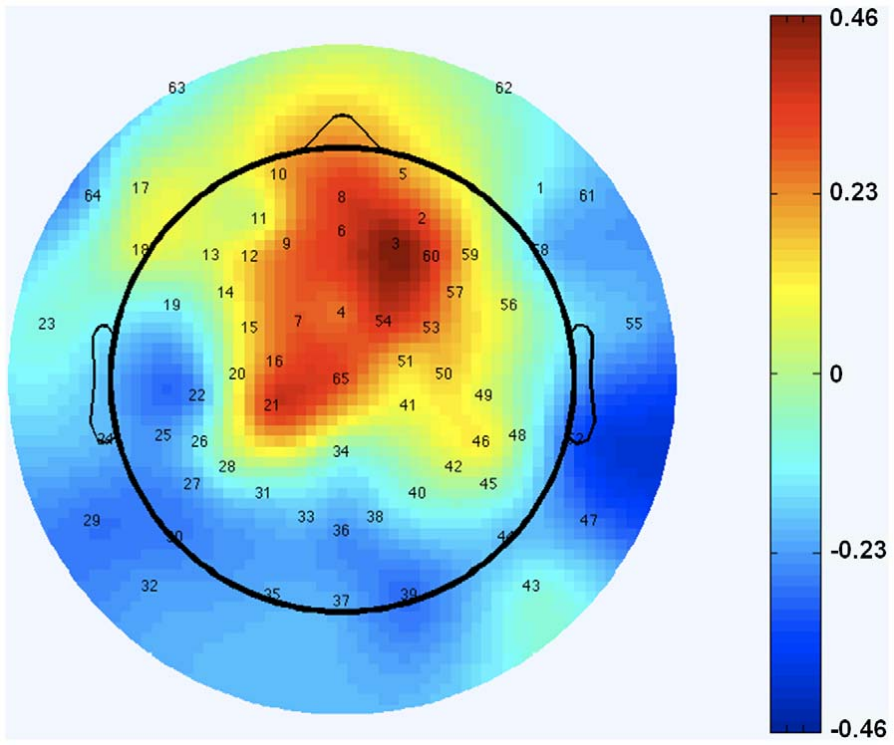
Predictive relation between early learning and 9-month neural activation of facial discrimination. Infants who learned more rapidly at one month of age showed greater discrimination in medial fronto-central activation to the mother's versus a stranger's face.

In addition to the assessment of social behaviors during the first year, face-evoked neural activity was also assessed at 9 months of age. To examine whether the rate of associative learning was related to neural activity of face discrimination, event-related potentials (ERPs) were recorded while infants viewed images of their mother's face and a stranger's face [27]. A difference wave of the Nc component between the mother's and stranger's face was computed for all electrode sites across the scalp. Greater associations between Learning Slope at one month of age and the Nc Amplitude Difference Score were observed over the medial fronto-central area (Figure 3), a region where the difference in Nc amplitude is expected to be greatest. These data indicate that infants who learned more rapidly at one month of age exhibit greater face discrimination between their mother and an unfamiliar female stranger.

Infant cognitive performance on the Mullen Scales of Early Learning (MSEL) [28] was assessed at 12 months of age. The MSEL is a standardized test used to assess 5 domains of functioning including gross motor, fine motor, visual reception, receptive language and expressive language, providing an overall Learning Composite Score. Infants in the current sample scored within the neurotypical range for all subscales as well as for overall cognitive abilities (Table 2). In contrast to social abilities, we found no significant correlation between 1-month Learning Slope and the Learning Composite Score or any subscale of the MSEL (*Ps*>.20; Table 2).

**Table 2:** Mullen Scales of Early Learning subscales.

*Mullen Subscales*	*T-Scores [Mean (SD)]*	*Correlation with 1-Month Learning [Pearson's r-value]*
Gross Motor	49.52 (14.39)	−.076
Fine Motor	51.74 (10.86)	−.061
Visual Reception	53.93 (9.89)	−.033
Receptive Language	45.57 (7.46)	.087
Expressive Language	51.50 (10.66)	−.005
Early Learning Composite	125.52 (15.50)	−.045

Mean T-scores and Pearson's correlations (*r*) with one-month learning slope.

## Discussion

The current study is the first to directly assess predictive relations between early associative learning and the emergence of social behaviors over the first year of life. Data reveal that individual differences in associative learning measured at one month of age relate to later social behavior across a variety of tasks and ages. Moreover, we discovered a significant neural correlate by demonstrating a relation between 1-month associative learning rate and neural responses to familiar versus unfamiliar face stimuli. These relations were specific to social behaviors and were not the result of individual differences in general cognition, suggesting that early associative learning may serve as a major building block for the development of social behavior.

These behavioral findings are consistent with the basic neurodevelopmental concept that both circuits and skills are built from simple to more complex, the latter being highly dependent upon the former [4]. It is clear, however, that the development and expression of social behaviors is complex, and cannot be fully explained by basic associative learning mechanisms. Nevertheless, the results of the current study provide the first evidence that associative learning should be considered as a major building block for early social development, and that early variability in learning serves as an important marker of individual differences in social development. Furthermore, the data here suggest that such variability may serve as a neurobiological signature of early detection for perturbations in social development (e.g., autism spectrum disorder).

## Methods

### Ethics Statement

The University of Maryland Institutional Review Board approved all procedures. Prior to data collection, written informed consent was obtained from a parent or caregiver during each visit.

### Participants

Seventy full-term healthy infants (34 males, 36 females) participated in the current study. Families were contacted by mail using commercially available lists of names and addresses compiled from local hospitals and infant registries. Prior to the laboratory visit at one month, parents completed a brief phone survey. Infants were excluded from participating in the study if they were born prior to 38 weeks of gestation, had reported birth complications or injury, serious illness, or diagnosed syndromic disorder.

The population of infants used in the current study was representative of the greater Washington, DC area with 51.4% Caucasian, 21.4% African American, 5.7% Hispanic, 4.3% Asian, and 17.1% mixed ethnicity. The infants' mothers were well educated with 41.1% completing a graduate degree, 38.6% completing a college degree, 4.3% completing a professional or trade certificate, and 14.3% completing a high school degree. Of the 70 families that visited the laboratory at one month, 10 did not return for follow-up visits for a permanent attrition rate of 15%. Of the remaining 60 families who continued to participate in the study, 55 returned for the 5-month visit, 51 returned for the 9-month visit, and 48 returned for the 12-month visit for attrition rates of 8%, 15%, and 21% respectively. Learning abilities of infants who participated in the follow-up assessments did not differ significantly from those who did not participate in the follow-up assessments (*t*s<1; *P*s>.2).

### Experimental Design

Individual differences in infant associative learning rates were assessed at 1 month of age and associated with heterogeneity in social behaviors measured during follow-up assessments at 5, 9, and 12 months of age (see Table 1 for details).

#### 1-Month Assessment

All infants were tested using a delay eyeblink conditioning paradigm while asleep. Six mm Ag/AgCl electrodes were positioned above and below the infant's left eye and one ground electrode was placed on the back of the infant's neck. The infant was then placed on his or her back in a bassinet with the head placed between two headphone speakers aligned directly with the infant's ears. Custom software controlled presentation of both the airpuff unconditioned stimulus (UCS; air compressor, San Diego Instruments, San Diego, CA) and auditory conditioned stimulus (CS). The airpuff was presented through tubing that was attached to a flexible plastic arm connected to the left speaker. The arm was positioned approximately 1 inch from the infant's left eyelid.

Trials consisted of the presentation of a 1000-ms, 1000-Hz tone that overlapped and co-terminated with a 100-ms airpuff, yielding a 900-ms delay interval. In each block of 10 trials, the 6^th^ trial was an airpuff-alone trial to test the somatosensory response and the 10^th^ trial was a tone-alone trial to test for a conditioned response. Stimuli were presented for a total of 15 blocks (120 tone-airpuff paired trials, 15 airpuff-alone trials, 15 tone-alone trials).

The raw electromyographic (EMG) signal was amplified using a custom bioelectric amplifier (SA Instruments, San Diego, CA) with a gain of 1000 Hz and filtered using high and low pass filters of 1 and 1250 Hz respectively. The amplified signal was digitized at a sampling rate of 512 Hz using a 12-bit A/D converter (±2.5 V input range) and Snap-Master data acquisition software (HEM Data Corporation, Southfield, MI). Prior to recording EMG from each participant, a 50 µV 10 Hz signal was input into the channel and the amplified signal was recorded for calibration purposes. The raw signal was processed and analyzed offline using the EMG Analysis System from James Long Company (Caroga Lake, NY). The signal was filtered digitally offline with a high-pass filter of 28 Hz, a low-pass filter of 250 Hz, and a digital band-stop filter (50–70 Hz) was used to remove 60-Hz noise. The signal was rectified and smoothed by using moving averages with a 20-ms window. Baseline EMG value was defined as the average activity recorded during the 20 ms prior to CS onset.

Tone-alone trials were examined for the occurrence of conditioned responses (CRs). Each trial was visually examined for the occurrence of an eyeblink response that was defined as a rapid deflection in the EMG signal that was at least 1 SD above the mean of the baseline and occurred between 800 and 1500 ms after tone onset. It should be noted that previous studies examining eyeblink conditioning in awake adults have defined CRs as blink responses that are at least 5 SD above a mean baseline [29], [30]. Preliminary analysis conducted on 10 infants showed that eyeblink responses did not reach this criterion even during puff-alone trials in which blink responses were the most discernible. This smaller magnitude eyeblink response in human infants may likely be attributed to the fact that the infants were tested while asleep. Therefore, the full range of eyelid movement from open to close was unable to be captured by the EMG recordings. In the current study, both tone-puff paired trials and tone-alone trials were visually examined for eyeblink responses. On trials in which a clear response occurred, it was determined that a 1 SD above baseline criterion was the most sensitive for both trial types. This criterion and smaller magnitude response in human infants is consistent with our previously reported data showing increased eyeblink responses during tone-puff trials compared to tone-alone trials [18]. The 1 SD criteria was the most sensitive to pick up blink responses that occurred during the tone-puff paired trials as well as the small blink responses that occurred during the tone-alone trials. Importantly, this criterion was also sensitive to the eyeblink conditioning paradigm as eyeblink responses were unlikely to be observed during earlier tone-alone trials compared to later tone-alone trials.

The percentage of CRs (%CR) across conditioning trials was used as the primary measure of learning and was computed across the 15 blocks in aggregates of 3 trials for a total of five 3-trial bins. To examine the relation between individual differences in learning and later social behaviors, the individual slope of the learning curve was computed for each infant by regressing %CR on Bin and used as the predictor variable in all subsequent analyses.

#### 5-month Assessment

During the 5-month assessment, two tasks were administered and maternal report on infant temperament was obtained. The tasks included were the peek-a-boo and puppets games [21] and infant temperament was assessed using the Infant Behavior Questionnaire (IBQ) [31].

The peek-a-boo and puppets tasks were specifically designed to elicit individual differences in displays of social positivity [21]. During the peek-a-boo game, the mother faced her infant and alternated between covering (1 s) and uncovering (4 s) her face with her hands. When her face was uncovered, she exclaimed “Peek-a-boo!” and displayed a large smile. The mother repeatedly covered and uncovered her face for 90 s.

During the puppets game [21], an experimenter sat on the end of the table closest to the infant and presented 2 puppets to the infant while the mother watched the puppets game from the opposite end of the table. While presenting the puppets, the experimenter used a scripted and standard dialogue. During the “puppet show”, the infant was tickled three times by the puppets: first by one, next by the other, and finally by both puppets simultaneously. The dialogue lasted approximately 90 s. After the puppet show, the experimenter placed the puppets on the table in front of the child for 30 s.

During the peek-a-boo and puppets games, behavioral positivity was coded separately including smiling intensity (0–3), intensity of vocalizations (0–3), and intensity of positive motor acts (0–2). The peek-a-boo game was divided into nine 10-s epochs for which the scores of each behavior were recorded. The puppets game was divided into a total of 5 epochs with four epochs equaling the time between each tickle and the last epoch occurring when the puppets were placed in front of the infant. In addition, presence of looking at the mother (0–1) was also coded during the puppets game as an index of social referencing. Two independent coders who were blind to the infants' 1-month learning abilities achieved sound inter-rater reliability on 20% of the data. Kappas ranged between .78 and .97 for behavioral positivity measured during the peek-a-boo game and .67 and .94 for the puppets game. The kappa for frequency of looking at the mother was .86. Individual measures for behavioral positivity were averaged across epochs for each game separately. The frequency of looking at the mother during the 5 epochs of the puppets game was computed. To obtain an overall Social Responsivity Score, behavioral positivity scores and frequency of social referencing were converted to *z* scores and averaged across both the peek-a-boo and puppets games. Of the 55 infants who participated in these tasks, 4 were unable to be coded due to technical difficulties with the video recording and were not used in the current analysis.

Maternal report of infant temperament was obtained using the IBQ [31]. The IBQ consists of 87 items that requires the mother to rate the frequency of her infant's behaviors that occurred within the last week along a 7-point Likert scale across a number of temperamental dimensions, including activity, soothability, distress to limitations, distress to novelty, and smiling/laughter. For the current study, only the smiling/laughter subscale was used. This subscale specifically assesses the infant's tendency to express smiling or laughter across social situations and includes items such as “When tossed around playfully, how often did the baby smile or laugh?” and “When introduced to a strange person, how often did the baby smile or laugh?” The IBQ was completed for 45 of the 55 infants.

#### 9-month Assessment

During the 9-month assessment, four tasks were administered in order to obtain individual differences in performance across several aspects of social behavior including social contingency detection, imitation, and social discrimination. These tasks include the still-face task [32] and the modified peek-a-boo game [21] to assess social contingency detection, imitation tasks [23], [24] to assess overall motor imitation, and the mother-stranger face discrimination task [27] to assess neural activity of social discrimination.

During the still-face task [32], the mother was instructed to sit directly across from the infant and interact with her infant as she normally would for 2 minutes (face-to-face interaction phase). Following this face-to-face interaction phase, the mother suddenly stopped the interaction and looked at her infant while maintaining a neutral expression for 2 minutes (still-face phase). During the final 2 minutes of the task, the mother tried to re-engage her infant in normal interaction (reunion phase). The Infant and Caregiver Engagement Phases (ICEP) [33] was used to code behaviors during all phases of the still-face task. Instances of protest and withdrawn behaviors were coded during 1-s epochs. Protest was characterized by displays of facial and bodily expressions of anger and withdrawn behavior was characterized by displays of sad facial expressions and wimpering/fussy vocalizations when disengaged from the mother. Sound inter-rater reliability was obtained on 20% of the data by two independent coders who were blind to the infants' one-month learning abilities. Kappas for protest and withdrawn behaviors were .94 and .85, respectively.

In order to obtain a negativity proportion score for each interaction phase, the frequency of protest and withdrawn behavior was summed separately for each phase and divided by the total duration of each phase. The classic still-face response is a decrease in positivity and increase in negativity from the face-to-face interaction phase to the still-face phase [34]. To capture this change in behavior, a still-face Social Contingency Detection score was computed for each infant and was defined as the difference in the proportion of negativity displayed during the still-face phase and the proportion of negativity displayed during the face-to-face interaction phase.

During the modified peek-a-boo game [21], the mother was instructed to stand behind a wooden screen containing 4-hinged doors while the infant sat in a highchair on the other side of the screen. The game consisted of 6 trials. During trials 1, 2, 3, and 6, the experimenter would ask the infant, “Where's mommy?” and then knock on and open the door to reveal a smiling mother who would exclaim, “Peek-a-boo!” During trials 4 and 5, the experimenter opened the door and revealed that the mother was absent. Infant behavior positivity during each trial was rated for smiling intensity (0–3), intensity of vocalizations (0–3), and intensity of positive motor acts (0–2). Two independent coders who were blind to the infants' 1-month learning abilities achieved sound inter-rater reliability on 20% of the data. Kappas ranged between .88 and .96 for the observed behaviors.

Behavioral scores were converted to *z*-scores and averaged separately across the trials in which the mother appeared behind the door and the trials in which the mother did not appear behind the door in order to obtain separate Social Positivity scores during mother-present and mother-absent trials. To examine individual differences in social contingency during the modified peek-a-boo task, a peek-a-boo Social Contingency Detection score was computed and defined as the difference between mother-present Social Positivity scores and mother-absent Social Positivity scores.

To examine imitation at 9 months, an experimenter presented the infant with four novel age-appropriate toys which were first described in studies conducted by Meltzoff [23] and Barr and colleagues [24]. These toys included a vertical wooden rectangle connected by a hinge to a larger rectangular wooden base, a black box that contained a button that could be pressed to sound a bell, a hollow plastic egg that contained beads inside, and a puppet that wore a mitten that contained a hidden bell attached to it. The experimenter presented each toy in a predetermined order and demonstrated how each toy was to be used – pushing the vertical block to lie flat, pushing the button on the box to ring the bell, shaking the plastic egg to produce a noise, and removing, shaking, and replacing the puppet's mitten to ring a bell. Prior to starting each presentation, the experimenter made sure the infant was attending to the toy by saying his or her name. The experimenter then demonstrated the action of each toy three times while keeping the toy just out of the infant's reach. Presentation order was counterbalanced across infants. Following a 10-min delay, the experimenter handed each toy to the infant in the same order of presentation and allowed the infant 30 seconds to imitate each of the target behaviors demonstrated to them by the experimenter. For additional details about the tasks, see [23], [24]. The task was scored for presence or absence of imitation (0–1). Two independent coders who were blind to the infants' 1-month learning abilities achieved sound inter-rater reliability on 20% of the data. Kappas ranged between .74 and 1 for task imitation. A total Imitation score was computed as the sum of the number of tasks correctly imitated by the infant. Of the 51 infants who returned to the laboratory at 9 months, 4 became upset during the imitation test and were unable to complete the task. Therefore, these infants were excluded from the current analysis.

During the mother-stranger face discrimination task [27], [35], infants were presented with color images of their mother's and a female stranger's face displaying neutral expressions while electroencephalographic (EEG) data was recorded. Prior to recording, a digital image of the mother was taken while the mother wore a gray scarf around her neck to conceal any clothing and stood in front of a gray screen. The mother's face was paired with a similar looking woman that was chosen from a database of faces of other mothers who participated in our study. The stranger's face was matched to the mother's face on hair color and style, face shape, ethnicity, and presence of eyeglasses.

Testing occurred while the infant sat on his or her mother's lap in a dimly lit room. Stimuli were presented using E-Prime software (Psychology Software Tools Inc., Pittsburgh, PA). Faces were presented on a black background and in the center of the screen. The computer monitor was 34 cm wide and 27 cm high. Infants viewed images at a distance of 60 cm. A camera mounted above the monitor allowed for simultaneous video recording of the infant's face during the experiment. The experimenter presented images during the experiment only when the infant was attending to the monitor. Trials were marked for deletion if the infant looked away during presentation of an image. Infants were presented 60 images of the mother's face and 60 images of the stranger's face. Faces were presented pseudo-randomly such that within every four presentations, the infant was randomly presented two images of the mother's face and two images of the stranger's face. Stimuli were presented for 500 ms followed by an inter-stimulus interval of at least 1000 ms during which time the screen was black with a white cross in the center.

ERPs were recorded using a 64-channel HydroCel Geodesic Sensor Net (Electrical Geodesics Inc., Eugene, OR). Signals were amplified using an EGI NetAmps 200 amplifier and sampled at 250 Hz with a band-pass filter of 0.1–100 Hz. Once the impedance values were reduced below 100 kΩ, data acquisition was started. EEG was recorded continuously and referenced to Cz, and after acquisition, data was re-referenced using an average reference.

Data were filtered offline using a 30-Hz lowpass and a 1-Hz highpass filter. Trials consisted of a 400-ms baseline period and 600-ms period following stimulus onset. Data were baseline corrected to the average voltage during the 400 ms prior to stimulus onset. Data were segmented and visually inspected for EOG and motion artifact. Data from individual sensors were rejected if there was artifact resulting from poor contact or movement. The entire trial was excluded if more than 15 sensors had been rejected, or if an eyeblink or other significant movement artifact had occurred. Of the trials that were not rejected, individual channels containing artifact were replaced using spherical spline interpolation. Individual subject averages were constructed separately for the mother and stranger faces.

Of particular interest to the current study was examination of the Nc component, a fronto-central negative deflection that reflects some aspect of visual attention [36]. Previous research has demonstrated that this component is larger when infants view the mother's versus a stranger's face [27]. Inspection of the grand-averaged waveforms revealed a well-defined Nc component that was strongest at the medial fronto-central sites and was subsequently analyzed within a time window 300–600 ms. In order to examine the correlation between Learning Slope and differences in Nc amplitude between the mother and stranger faces, a difference wave was computed and the mean amplitude of the difference wave at each electrode site was correlated with individual infant's Learning Slope scores. Correlation *r*-values between Learning Slope and the Nc mean amplitude difference at each electrode site were subsequently submitted to EEGLAB software [37] to obtain a topoplot of the scalp showing the distribution of the Learning Slope-Nc difference correlations (Figure 3). Of the 51 infants who participated during the 9-month laboratory assessment, 7 were excluded from ERP analysis due to either incorrect net placement (*N* = 4) or becoming too upset and unable to finish the task (*N* = 3). These infants were excluded from the current ERP analysis.

#### 12-month Assessment

During the 12-month assessment, one task was administered to examine infant joint attention and one task was administered to examine infant cognitive abilities. The tasks included were the Early Social Communication Scale (ESCS) [26] to assess joint attention and the Mullen Scales of Early Learning (MSEL) [28] to assess cognition.

The ESCS [26] is a semi-structured assessment that elicits joint attention and behavioral requests in infants and young children. During the assessment, the experimenter presented attractive toys and objects to the infant while the infant was seated on his or her mother's lap. The duration of the assessment was approximately 15–20 minutes. Infant's behavior was coded for instances of responding to joint attention (RJA) and initiating joint attention (IJA). RJA refers to instances in which an adult draws the infant's attention toward a specific object by gazing or pointing at the object and the infant subsequently looks at the object of interest. IJA refers to instances in which the infant draws the adult's attention toward a specific object and continues to monitor the adult's attention toward the object be repeatedly looking between the adult's line of vision and the object. Two independent coders who were blind to the infants' 1-month learning abilities achieved sound inter-rater reliability on 20% of the data and intra-class correlation *r*s were .767 and .921 for IJA and RJA, respectively. A total Joint Attention score was computed by averaging standardized scores (*z*-scores) of IJA and RJA.

The MSEL [28] is a standardized cognitive test for children 0–69 months of age and is used to assess 5 domains of functioning including gross motor, fine motor, visual reception, receptive language, and expressive language as well as give an overall learning composite score (Table 2).

### Statistical Analysis

Repeated measures ANOVA with Bin as the within measure was used to determine if infants' learning increased across the course of the experiment. Pearson's correlations were used to determine if there were significant relations between individual differences in 1-month learning and 5-, 9-, and 12-month social outcome measures and 12-month cognition.
